# ﻿New species of *Plesioaxymyia* Sinclair (Diptera, Axymyiidae) from the Palaearctic Region, including an updated molecular phylogeny of the family

**DOI:** 10.3897/zookeys.1236.148218

**Published:** 2025-05-05

**Authors:** Alexei Polevoi, Nikola Burdíková, Jan Ševčík

**Affiliations:** 1 Forest Research Institute, 185035, Pushkinskaya 11, Petrozavodsk, Russia Forest Research Institute Petrozavodsk Russia; 2 Department of Biology and Ecology, Faculty of Science, University of Ostrava, Chittussiho 10, CZ-710 00 Ostrava, Czech Republic University of Ostrava Ostrava Czech Republic

**Keywords:** Biology, distribution, lower Diptera, Nematocera, new species, Russia, systematics

## Abstract

The family Axymyiidae includes four extant genera and nine species known from the Holarctic and Oriental regions, with only one species *Mesaxymyiakerteszi* (Duda, 1930) occurring in Europe. The genus *Plesioaxymyia* Sinclair, 2013 was first discovered in Alaska in 1962, but officially described only many years later. Up to now, the genus included one species *Plesioaxymyiavespertina* Sinclair, 2013, known from two female specimens from western North America. During the study of the Diptera fauna in Paanajarvi National Park (Northwest Russia), one female specimen of *Plesioaxymyia*, was found. It differs from the Nearctic *P.vespertina* by details of its female terminalia and is recognized as a new species, herein described as *Plesioaxymyiaimprevista***sp. nov.** The biology and geographic distribution of *Plesioaxymyia* is briefly discussed. The phylogenetic position of the genus, along with the family Axymyiidae is analyzed in the light of new molecular data, including sequences of three mitochondrial (ribosomal 12S and 16S and protein-encoding COI) and three nuclear genes (ribosomal 18S and 28S, protein-encoding CAD) for 72 terminal taxa. Axymyiidae is recovered as a monophyletic group with closest relatives in the infraorder Culicomorpha and *Plesioaxymyiaimprevista***sp. nov.** representing the sister taxon to all the other species of Axymyiidae included in the analysis.

## ﻿Introduction

The family Axymyiidae is often referred to as “enigmatic” in the literature (Wihlm and Courtney 2011; Blagoderov and Lukashevich 2013; Fitzgerald and Wood 2014). This small group of flies includes four extant genera and nine species known in the Holarctic and Oriental regions (Fitzgerald and Wood 2014). Additionally, eight species in four extinct genera have been described from the Jurassic or Early Cretaceous deposits of Asia (Blagoderov and Lukashevich 2013; Shi et al. 2013). Only one species, *Mesaxymyiakerteszi* (Duda, 1930), is so far known from Europe. This is an extremely rare taxon, with a few recent records from eastern Slovakia (Martinovský and Roháček 1993) and Northwest Russia (Polevoi et al. 2018).

As it is known so far, all immature records of Axymyiidae are associated with dead wood. Extremely specialized larvae live inside very wet, often submerged, logs of various tree species (Wood 1981; Krivosheina 2000). The systematic position of this family is still uncertain (see Blagoderov and Lukashevich 2013; Sinclair 2013; Fitzgerald and Wood 2014 for reviews). It is often placed in or as a sister group to Bibionomorpha, but close relationships with this infraorder are not always supported by reconstructions, based on molecular data. In the comprehensive study by Wiegmann et al. (2011), Axymyiidae were placed within Bibionomorpha sensu lato, as a sister group to Bibionomorpha sensu stricto, whereas in other studies this family was represented as a sister group to Culicomorpha (Bertone et al. 2008; Ševčík et al. 2016) or in an unresolved polytomy (Zhang et al. 2023).

During studies of the Diptera fauna in Paanajarvi National Park (Russia, Karelia), an unusual looking female specimen was collected, evidently belonging to the family Axymyiidae. Preliminary examination showed that it was not a representative of any previously known Palaearctic genus, but accords well with the genus *Plesioaxymyia* Sinclair, which includes one North American species, *P.vespertina* Sinclair, 2013. Being morphologically very similar, the Karelian specimen differs from *P.vespertina* by characters of its terminalia. Considering the general rarity of the family Axymyiidae and the evident importance of the new record we feel it necessary to describe the new species and not wait for additional material. Moreover, we could not miss an opportunity to re-evaluate the phylogenetic position of *Plesioaxymyia* and Axymyiidae as a whole, based on new molecular data for extant species.

## ﻿Material and methods

**Figure 1.**
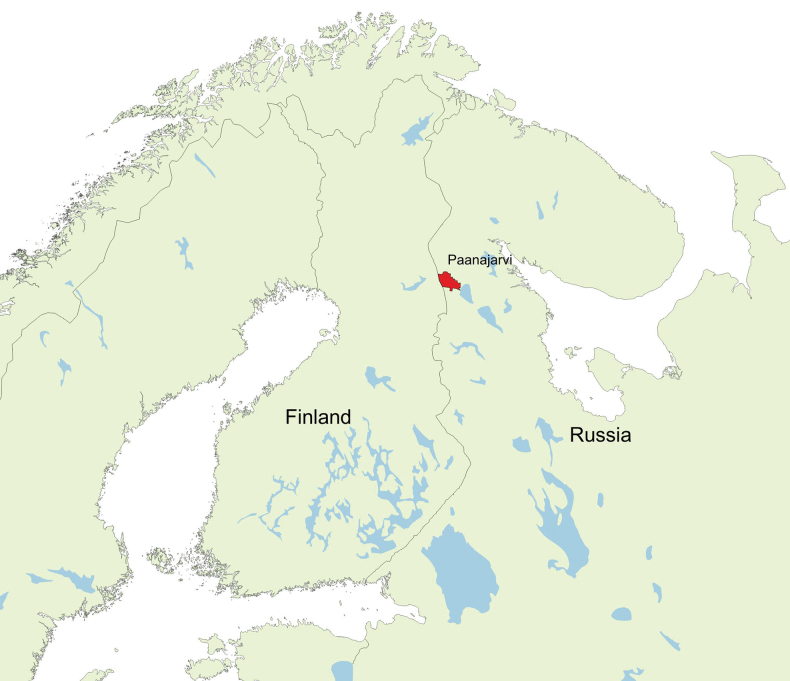
Location of Paanajarvi National Park, Karelia, Russia.

The type specimen was collected in Paanajarvi National Park, located in the northwestern part of the Republic of Karelia, Russia (Fig. 1). The park was established in 1992, and presently occupies a territory of over 104,000 ha, mostly covered with natural coniferous forests (Bizhon and Systra 1996; Timofeeva and Kutenkov 2009). In 2021, we used three Malaise traps as part of an insect inventory program in the territory of the park. The *Plesioaxymyia* specimen was collected with a trap set in a patch of spruce- and pine-dominated forest of *Vacciniummyrtillus* type, in the vicinity of the abandoned village of Vartolambina. The operation period of the trap was from June 1–27, which corresponds to late spring. Willows and some early-season flowers (e.g., *Tussilago*) were in blossom, and remnants of snow still could be found in shady places.

The trap residue was initially kept in 70% alcohol, and one Axymyiidae specimen was recognized during a preliminary inspection in the laboratory. For a detailed study, the specimen was dried by xylol and amyl acetate baths (Achterberg 2009). Terminalia were detached and macerated in KOH for 24 hours, then neutralized in acetic acid, washed in 70% alcohol, and transferred to glycerine. Finally, terminalia were placed in glycerine vial and pinned together with the rest of the specimen. The holotype is stored in the collection of the Zoological institute, St. Petersburg, Russia (**ZISP**).

Images of the habitus and wing were taken with a Leica MZ 9.5 stereo microscope and those of the terminalia with a Leica DM1000 compound microscope, both supplied with a LOMO MC6.3 camera. Z-stacked image series were combined using Helicon Focus v. 8.2.0 software, and final plates prepared with GIMP. The morphological terminology follows Sinclair (2013). The distribution map was created using the online tool SimpleMappr, available at https://www.simplemappr.net/.

Molecular methods principally follow those described in Ševčík et al. (2016). A total of 72 terminal taxa are included in the dataset (Appendix 1). Most of the specimens used in this study were collected by Malaise traps during the years 2000–2022. Some sequences were taken from the GenBank database. The material used in the molecular study was stored in ethanol (70% to 96%) or pinned. For DNA extraction, we used a NucleoSpin Tissue Kit (Macherey-Nagel, Düren, Germany) following the manufacturer’s protocol. PCRs (total volume = 20 μl) were performed using specific primers for sequences of three mitochondrial (ribosomal 12S and 16S and protein-encoding COI) and three nuclear genes (ribosomal 18S and 28S, protein-encoding CAD). The primers used for PCR amplifications and sequencing are listed in Table 1.

**Table 1.** Primers used for PCR amplification and sequencing of the mitochondrial 12S, 16S and COI genes and nuclear 18S, 28S and CAD genes.

Gene fragment	Direction	Primer sequences (5´→ 3´)	Source
12S	F	CTGGGATTAGATACCCTGTTAT	Koufopanou et al. 1999
	R	CAGAGAGTGACGGGCGATTTGT	Koufopanou et al. 1999
	F	TACTATGTTACGACTTAT	Kambhampati and Smith 1995
	R	GCCAGCATTTGCGGTTATAC	M. Žurovcová lab., České Budějovice, Czech Republic
16S	F	TAATCCAACATCGAGGTC	Roháček et al. 2009
	R	CGAAGGTAGCATAATCAGTAG	Roháček et al. 2009
	F	CGCCTGTTTATCAAAAACAT	Palumbi et al. 1991
	R	CCGGTCTGAACTCAGATCACGT	Palumbi et al. 1991
18S	F	AACCTGGTTGATCCTGCCAGT	Katana et al. 2001
	R	TGATCCTTCTGCAGGTTCACCTACG	Katana et al. 2001
	F	AGATACCGCCCTAGTTCTAACC	Campbell et al. 1995
	R	GGTTAGAACTAGGGCGGTATCT	Campbell et al. 1995
28S	F	AGAGAGAGAGTTCAAGAGTACGTG	Belshaw and Quicke 1997
	R	TAGTTCACCATCTTTCGGGTC	Laurenne et al. 2006
	F	ACCCGCTGAATTTAAGCAT	Dayrat et al. 2001
COI	F	GGTCAACAAATCATAAAGATATTGG	Folmer et al. 1994
	R	TAAACTTCAGGGTGACCAAAAAATCA	Folmer et al. 1994
CAD	F	GGNGTNACNACNGCNTGYTTYGARCC	Moulton and Wiegmann 2004
	R	TTNGGNAGYTGNCCNCCCAT	Moulton and Wiegmann 2004
	F	ACNGAYTAYGAYATGTGYGA	Moulton and Wiegmann 2004
	R	TCRTTNTTYTTWGCRATYAAYTGCAT	Moulton and Wiegmann 2004

All amplified products were purified using a Gel/PCR DNA Fragments Extraction Kit (Geneaid, New Taipei City, Taiwan) following the manufacturer’s protocol. Purified products were sequenced by Macrogen Europe (Netherlands) or Eurofins Genomics (Germany). The sequences were assembled and edited in Sequencher v. 5.0 (Gene Codes Corporation, Ann Arbor, MI, USA) or SeqTrace v. 0.9.0 (Stucky 2012). GenBank accession numbers for all the sequences are listed in the Appendix 1. All sequences were checked with the NCBI database using the BLAST and in single-gene trees to avoid possible contamination or other inappropriate results.

Alignments of all genes were created using MAFFT v. 7 on the MAFFT server (http://mafft.cbrc.jp/alignment/server/). The resulting alignments were visually inspected and manually refined in BioEdit v. 7.2.5 (Hall 1999) when necessary. All unreliably aligned regions of rRNA genes were removed in the program GBLOCKS v. 0.91b (Castresana 2000); with conditions set as follows: allow smaller blocks, allow gap positions within the final blocks, allow less strict flanking positions and do not allow many contiguous non-conserved positions, or in ClipKIT v. 2.2.2 (Steenwyk et al. 2020) using the -gappy option with a threshold of 0.7. The third positions of COI gene were excluded from the subsequent analyses using software DAMBE (Xia and Xie 2001). The alignments were concatenated using FASconCAT v. 1.0 (Kück and Longo 2014). The final data matrix consisted of 5419 characters: 12S – 301 bp, 16S – 287 bp, 18S – 1985 bp, 28S – 1005 bp, COI – 436 bp (third positions removed) and CAD – 1405 bp.

The final concatenated dataset was partitioned by gene and codon position and subsequently analysed using the maximum likelihood (ML) method. Analyses were conducted using IQ-TREE v. 1 (Nguyen et al. 2015) on the IQ-TREE web server (Trifinopoulos et al. 2016). Best-fitting substitution models were chosen automatically by the IQ-TREE software: 12S – TVM+F+I+G4; 16S – TVM+F+I+G4; 18S – GTR+F+I+G4; 28S – TVM+F+I+G4; CAD_1 – TN+F+I+G4; CAD_2 – SYM+I+G4; CAD_3 – GTR+F+I+G4; COI_1 – TIM2+F+I+G4; COI_2 – TIM3+F+I+G4; without free-rate heterogeneity. Branch supports were evaluated using 1000 ultrafast bootstrap (Hoang et al. 2018). All other settings were left as default. The node support values are given in the form of ultrafast bootstrap (= ufboot). The resulting phylogenetic tree (consensus trees) was visualized using the Interactive Tree of Life (iTOL v. 7.0; Letunic and Bork 2024). A species of Mecoptera, *Boreushyemalis* (Linnaeus, 1767), was used as a root.

## ﻿Results

### ﻿Family Axymyiidae

#### ﻿ Plesioaxymyia imprevista

Taxon classificationAnimaliaDipteraAxymyiidae

Polevoi sp. nov.

FA16C5BE-26EC-510C-BC4A-857827D0D5E8

https://zoobank.org/F101EC9C-CA50-40E2-ABA1-B631CAD5F331

Figs 2A–C , 3A–C

##### Specimens examined.

***Holotype.*** Russia • ♀; Karelia, Paanajarvi National Park, Vartolambina; 66.246°N, 30.555°E; 80 m a.s.l.; 1–27 Jun. 2021; A. Protasova leg.; Malaise trap; GenBank: accession numbers PV036313, PV036316, PV036319, PV036317, PV035246, PV037681; ZISP, INS_DIP_0001011.

##### Differential diagnosis.

**Figure 2.**
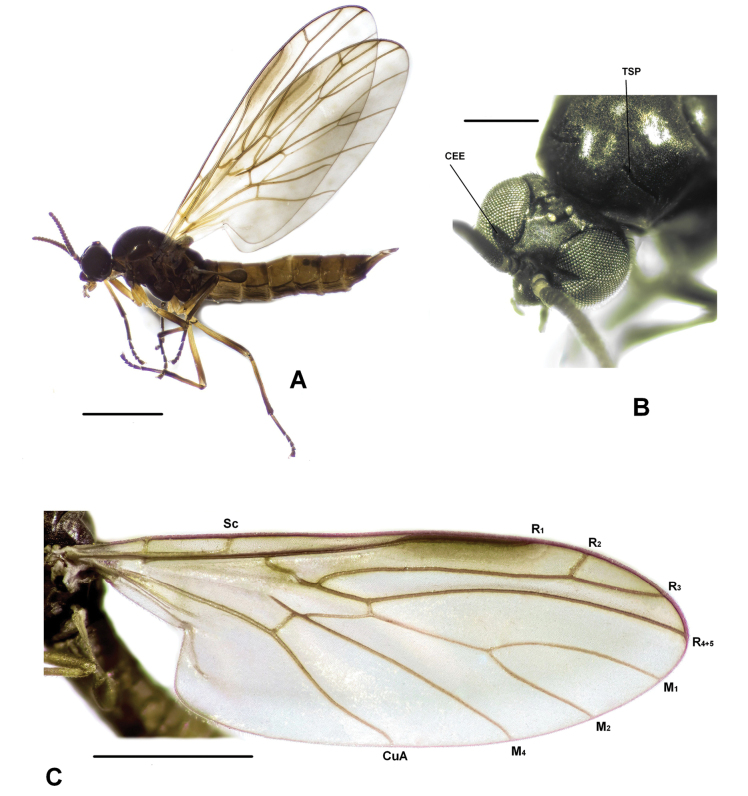
*Plesioaxymyiaimprevista* sp. nov., female holotype **A** habitus, lateral view **B** head and thorax, oblique anterodorsal view **C** wing. Abbreviations: CEE – triangular excision of compound eye; CuA – anterior branch of cubital vein; M_1,2,4_ – medial veins; R_2,3,4+5_ – radial vein; Sc – subcostal vein; TSP – transverse shiny patch of mesonotum. Scale bars: 1 mm (**A, С**); 0.3 mm (**B**).

Medium-sized, dark brown species (Fig. 2A); wings hyaline with dark elongated pterostigma, covering apical half of vein R_1_; legs yellowish-brown with darkened tarsi, femora and tibiae darkened apically. Similar to *Plesioaxymyiavespertina*, from which it is distinguished by details of female terminalia: sternite 8 with smoothly rounded dorsoapical margin (forming somewhat protruding dorsoapical corner in *P.vespertina*) and completely reduced basal segment of cerci (distinct in *P.vespertina*).

##### Description.

***Head.*** Head dark brown. Face sunken, clypeus convex, mouthparts brown. Palpus brown, 5-segmented, with short (almost hidden) first segment and broadened third segment. Compound eye densely covered with short ommatrichia; divided by fine groove into upper and lower hemispheres of unequal size and with deep triangular excision opposite antennal base (Fig. 2B). Three ocelli arranged in equilateral triangle, placed on elevated tubercle. Frons with a few brownish hairs above antennal bases and between ocellar triangle and compound eye. Posterior part of head with numerous brownish hairs. Antenna 16-segmented, brown; pedicel yellowish apically; middle flagellomeres about twice as wide as long.

***Thorax.*** Mesonotum dark brown, thinly dusted, lacking any larger setae but covered with tiny yellowish hairs; a pair of narrow transverse shiny patches present along prescutal suture (Fig. 2B); prescutum yellowish laterally. Scutellum yellowish-brown, strongly convex, with short hairs along posterior margin. Pleura brown.

***Wing.*** Wing length 4.08 mm. Wing hyaline with light brownish tinge (Fig. 2C); brown elongated pterostigma occupies apical half of R_1_. Costa hardly produced beyond R_4+5_. Sc curved into costa proximally to Rs; Sc-r reduced. Rs with kink; R_2+3_ forked well beyond apex of R_1_, R_2_ deviates in slightly obtuse angle. Crossvein r-m perpendicular to R_4+5_. M_1+2_ branching slightly before apex of R_1_; section of M-stem distal to r-m about as long as M_1_, longer than M_2_ and about twice as long as its section proximal to r-m. M_4_ straight; CuA distinctly sinuous. CuP short, scarcely reaching beyond posteromedial angle of wing. Anal lobe well-developed. Macrotrichia on wing veins not visible. Halter brown.

***Legs.*** Coxa, trochanters, femora and tibiae yellowish-brown; all femora and tibiae darkened apically; tarsi brownish. Hind tibia slightly curved in middle, with brush of bristly hairs posteroapically. Ratio of basitarsus to tibia: bt1:t1 – 0.45, bt2:t2 – 0.43, bt3:t3 – 0.30. Tibial spurs not developed.

**Figure 3.**
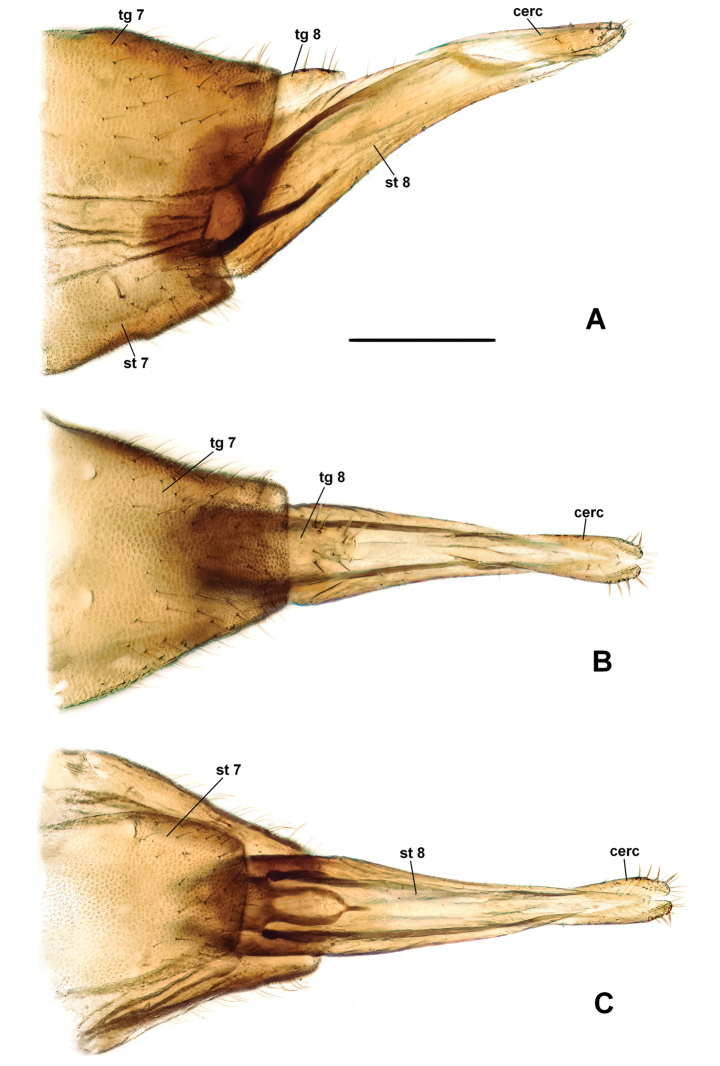
*Plesioaxymyiaimprevista* sp. nov., female terminalia **A** lateral view **B** dorsal view **C** ventral view. Abbreviations: cerc—cercus; st 7, 8—sternites; tg 7, 8—tergites. Scale bars: 0.2 mm (**A–C**).

***Abdomen.*** Abdomen brown. Tergites 1–7 with sparse hairs posteriorly. Terminalia (Fig. 3) brown. Tergite 8 approximately one-third length of sternite 8, rounded and setose apically; sternite 8 lengthened, tapered apically, with sparse short hairs. Cercus one-segmented; basal segment not developed; apical segment about 4 times longer than wide, bearing ca. 10 setae.

##### Etymology.

The species epithet is from the Latin *imprevistus* (unexpected, unforeseen), stressing that the finding of this species in Northwest Russia was a real surprise.

##### Distribution.

The species is currently known only from the type locality in Russian Karelia (Northwest Russia).

##### Biology.

The adult was collected with a Malaise trap set in *Vacciniummyrtillus* type pine- and spruce-dominated forest. The larval biology is unknown.

### ﻿Key to the Holarctic species of *Plesioaxymyia*, females

**Table:** 

1	Sternite 8 forming somewhat protruding dorsoapical corner in lateral view, basal segment of cerci distinct (Sinclair 2013, figs 4, 5)	***P.vespertina* Sinclair, 2013**
–	Sternite 8 with smoothly rounded dorsoapical margin in lateral view, basal segment of cerci completely reduced (Fig. 3A, B)	***P.imprevista* sp. nov.**

### ﻿Molecular data

**Figure 4.**
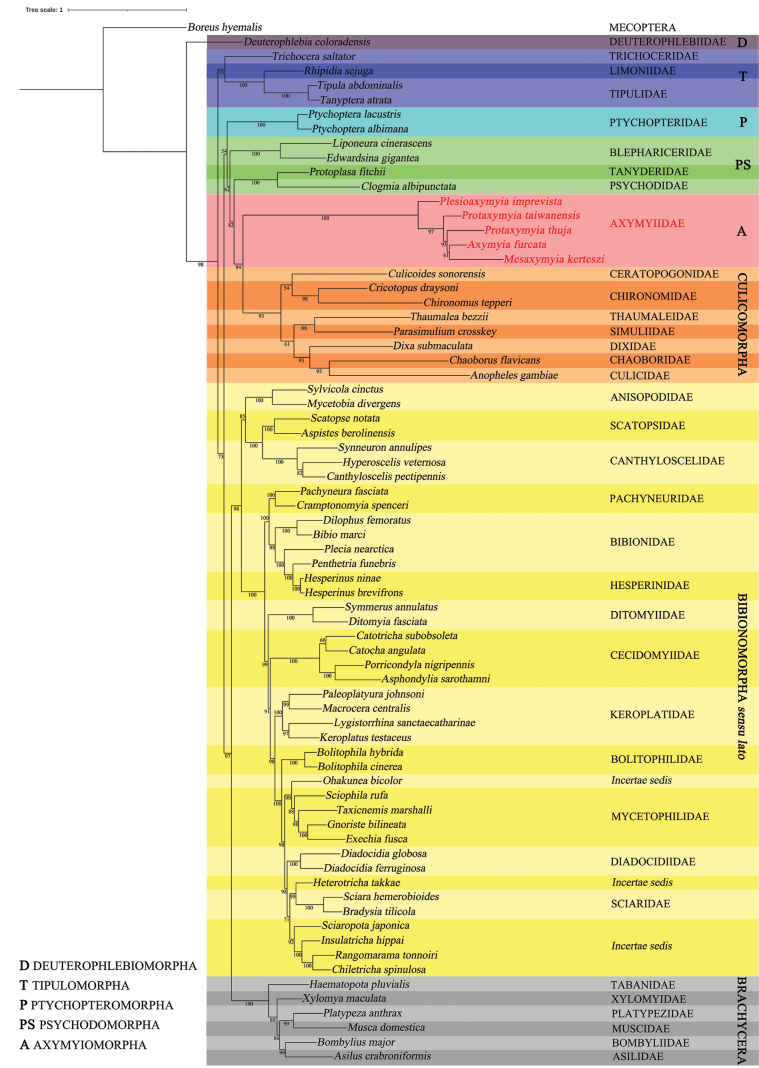
Maximum likelihood hypothesis (IQ-TREE) for relationships among selected taxa of lower Diptera based on DNA sequence data /12S, 16S, 28S, 18S, COI (third positions removed) and CAD/, 5419 characters. Support numbers refer to ultrafast bootstrap values (ufboot) over 50.

The family Axymyiidae, represented in this dataset by five species from all four extant genera, is recovered as monophyletic with maximum support (ufboot = 100) (see Fig. 4). The new species described in this paper, *Plesioaxymyiaimprevista* sp. nov., represents the sister taxon to all other species of Axymyiidae included in the dataset. Surprisingly, two species of *Protaxymyia* are not recovered as sister taxa. Instead, *Protaxymyiathuja* Fitzgerald & Wood, 2014 is sister species (ufboot = 95) to the branch including *Axymyiafurcata* McAtee, 1921 and *Mesaxymyiakerteszi*. The sister relationship of the latter two species is, however, weakly supported (ufboot = 61).

The closest relative of the family Axymyiidae appears to be the infraorder Culicomorpha, although their sister relationship is only moderately supported (ufboot = 84). The well-supported clade (Tanyderidae + Psychodidae), represented by *Protoplasafitchii* Osten Sacken, 1859 and *Clogmiaalbipunctata* (Williston, 1893), forms a sister group to the clade (Axymyiidae + Culicomorpha), also with moderate support (ufboot = 82).

The infraorder Bibionomorpha sensu stricto is shown to be monophyletic with high support (ufboot = 100), as well as Bibionomorpha sensu lato (ufboot = 98), including also Anisopodidae, Canthyloscelidae and Scatopsidae. The sister clade to Bibionomorpha is the infraorder Brachycera, altogether forming the well-supported group Neodiptera (ufboot = 97), which is sister clade to the large group of aquatic or semi-aquatic lower Diptera formed by the families Ptychopteridae, Blephariceridae, Tanyderidae, Psychodidae, and Axymyiidae and the families of the infraorder Culicomorpha.

## ﻿Discussion

### ﻿Biology and distribution

Some species of the family Axymyiidae can be rather abundant in suitable places, at least in respect of the density of larvae (Krivosheina 2000; Wihlm and Courtney 2011). However, representatives of the genus *Plesioaxymyia* apparently are not among them. The Nearctic *P.vespertina* was searched for repeatedly in the location where it was first found (Sinclair 2013). It was encountered again only 50 years later, and far to the south of the earlier known locality. The Paanajarvi area in Russian Karelia has been known for its relatively well-studied entomofauna since the middle of 20^th^ century (Viramo 1998; Yakovlev et al. 2000), but during this time no Axymyiidae came into any entomologist’s view. All known records of *Plesioaxymyia*, both in North America and Russian Karelia, look to be accidental, which may be the result of extremely small populations or a cryptic lifestyle.

**Figure 5.**
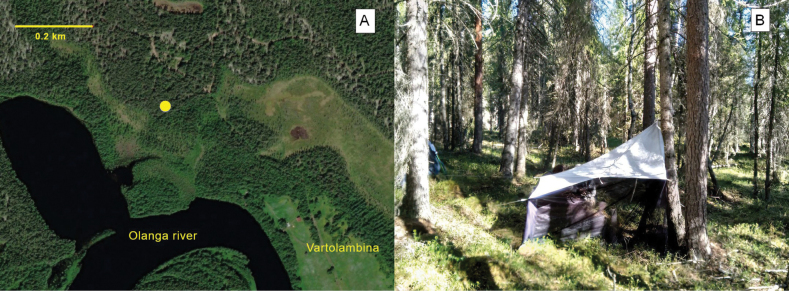
Collecting biotope of *Plesioaxymyiaimprevista* sp. nov. **A** position of Malaise trap (yellow circle), displayed on a satellite image (https://www.bing.com/maps) **B** general view of the biotope.

Adults of Axymyiidae usually can be found in shady habitats, near rivers, rivulets or other water bodies, close to woody substrates where the larvae develop (Krivosheina 2000; Wihlm and Courtney 2011). The collecting localities of *P.vespertina* in North America well meet these conditions (Sinclair 2013); however, *P.imprevista* sp. nov. was found in a patch of relatively dry coniferous forest (Fig. 5). The patch is bordered from the north by *Cladonia* type pine stands, and the only available water body around is the Olanga River, about 200 m to the south. Considering unsuccessful attempts to collect immature stages at the Alaskan and Mt. Rainier localities, Sinclair (2013) suggested that the larva of *P.vespertina* may reside in wet litter or decomposing fungi. In Paanajarvi, potentially suitable dead wood sources were totally absent close to the trap location. The distance from the river, however, does not look unreachable (even if Axymyiidae are considered weak fliers), so the wet dead wood, potentially available along the riverbank, cannot be excluded as a larval substrate. In general, the biology of *Plesioaxymyia* remains almost unknown. For now, we only can outline the flight activity period and preferred habitats, while other details of the life history are yet to be discovered.

**Figure 6.**
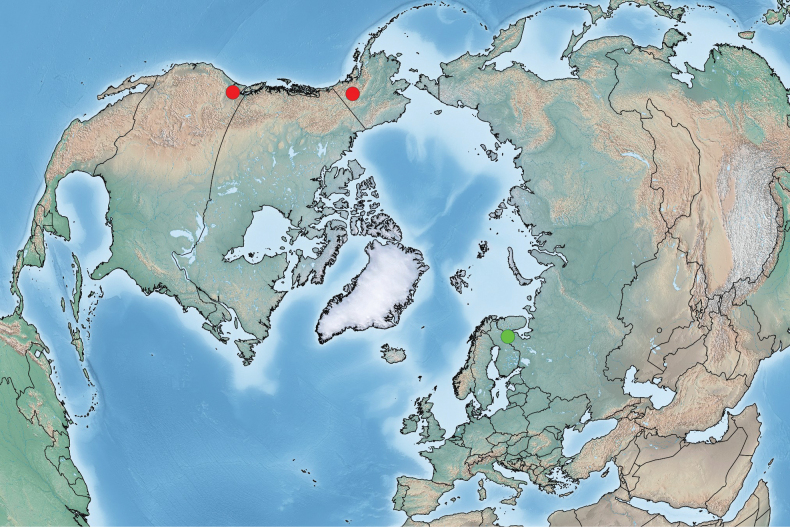
Distribution of the genus *Plesioaxymyia* in the Holarctic Region. *Plesioaxymyiavespertina* is indicated by red circles and *P.imprevista* sp. nov. by a green circle.

The current range of *Plesioaxymyia* comprises the western Nearctic and western Palaearctic (Fig. 6). It is highly likely that the current disjunct pattern is just a result of insufficient knowledge. Considering the occurrence of the genus at relatively high latitudes (or high altitudes in more southern regions), a circumpolar, possibly boreo-alpine, distribution can be suggested, and further findings from at least the northern regions of East Russia can be expected.

### ﻿Molecular phylogeny

The position of Axymyiidae as a sister group of Culicomorpha was initially revealed by Bertone et al. (2008), whose reconstruction was based on four nuclear gene markers and one sequenced taxon, namely *Axymyiafurcata*. It was rendered together with *Nymphomyiadolichopeza* Courtney, 1994 (Nymphomyiidae) with relatively low branch support (56%). The authors themselves considered this placement to be ambiguous, suggesting that it may have been influenced by the long-branch attraction effect. The subsequent study by Ševčík et al. (2016), which was based on six gene markers, focused on Bibionomorpha. However, it also included two species of Axymyiidae (*Axymyiafurcata* and *Protaxymyiathuja*) among numerous taxa in the broad outgroup. In this study, the sister group relation to Culicomorpha was identified again, though with similarly low support. The recent reconstruction, based on the complete mitochondrial genome (Zhang et al. 2023), included one taxon (*Protaxymyia* sp.) and did not demonstrate any clear relations for Axymyiidae, whose position appeared in an unresolved polytomy.

Sinclair (2013) proposed that *Plesioaxymyia* is sister group to the remaining Axymyiidae based on morphological characters. This hypothesis is now corroborated by genetic data. However, the grouping of other species deviates from expectations. Notably, *Protaxymyiathuja* is not rendered as sister taxon to *P.taiwanensis* Papp, 2007, being placed closer to species described in different genera (*Axymyiafurcata* and *Mesaxymyiakerteszi*).

The distinction between the genera of Axymyiidae (except the very peculiar *Plesioaxymyia*) is not clearly defined. Separation of adults largely relies on wing characters (Krivosheina 2000; Zhang 2010; Shi et al. 2013); however, with the accumulation of new materials, it has become evident that wing venation is not stable and its significance in the generic classification of Axymyiidae, including extinct genera, should be re-evaluated (Martinovský and Roháček 1993; Blagoderov and Lukashevich 2013; Fitzgerald and Wood 2014). The importance of larval and pupal characters (Krivosheina 2000; Fitzgerald and Wood 2014) is similarly unclear, given that preimaginal stages of several species have not yet been discovered.

## ﻿Conclusion

Our reconstruction of the molecular phylogeny is the first to incorporate representatives of all extant genera of Axymyiidae, although this still represents only approximately half of the known species. Axymyiids constitute a relatively isolated group, with the Culicomorpha families representing its closest evolutionary relatives. Both groups share an aquatic or semi-aquatic larval habitat. The sister group relation of Axymyiidae to Culicomorpha is in agreement with the findings of Bertone et al. (2008) and Ševčík et al. (2016) and is now evidenced with much better (ufboot = 84) support. While this may not yet be considered as strongly reliable, it is assumed that it could be even better supported if more taxa were included.

The current, unexpected placement of the species in relation to the Axymyiidae genera may be an artefact caused by the incomplete DNA data for *Mesaxymyiakerteszi* and *Axymyiafurcata*. However, new molecular data provide additional background for the necessity of a revised generic classification of Axymyiidae. It is likely that such a revision will not be possible until details of the morphology of preimaginal stages and, preferably, also genetic data on all extant species are available.

## Supplementary Material

XML Treatment for Plesioaxymyia imprevista

## ﻿Additional information

### Author ORCIDs

Alexei Polevoi https://orcid.org/0000-0003-2932-9574

Nikola Burdíková https://orcid.org/0009-0009-0735-4411

Jan Ševčík https://orcid.org/0000-0001-7174-593X

### Data availability

All of the data that support the findings of this study are available in the main text or Supplementary Information. Sequence data used in this article are available in the GenBank Nucleotide Database at https://www.ncbi.nlm.nih.gov/genbank.

## ﻿Appendix 1

**Table A1.** List of specimens used for the phylogenetic analysis, with GenBank (GB) accession numbers.

Species	Voucher code	Sampling locality and year	12S	16S	18S	28S	COI	CAD
MECOPTERA								
*Boreushyemalis* (Linnaeus, 1767)	borhym	from GB	GAYK00000000.2					
DIPTERA								
**Anisopodidae**								
*Mycetobiadivergens* Walker, 1856	OUT39	USA, 2014	KP288703	KP288735	KP288781	KP288718	KT316870	FJ040587
*Sylvicolacinctus* (Fabricius, 1787)	SylCin	Czech Republic, 2016	PV156352	PV156360	PV206766	PV230690	PV153731	PV158022
**Axymyidae**								
*Axymyiafurcata* McAtee, 1921	axyfur	from GB	n/a	n/a	n/a	KC177639	MG112467	KC177110
*Mesaxymyiakerteszi* (Duda, 1930)	BA466	Russia, 2016	PV036314	PV036315	n/a	PV036318	PV035247	n/a
*Plesioaxymyiaimprevista* sp. nov.	BA187	Russia, 2021	PV036313	PV036316	PV036319	PV036317	PV035246	PV037681
*Protaxymyiataiwanensis* Papp, 2007	ProTai	Taiwan, 2019	PV156350	PV156358	PV206762	PV230687	PV153729	PV158019
*Protaxymyiathuja* Fitzgerald &Wood, 2014	OUT38	USA, 2014	KP288702	KP288734	KP288780	KP288817	KT316869	KX453744
**Blephariceridae**								
*Edwardsinagigantea* Zwick, 1977	edgi	from GB	KC177470	KC177460	KC177283	KC177655	KC192960	FJ040624
*Liponeuracinerascens* Loew, 1844	LipCin	Slovakia, 2021	PV156349	PV156357	PV206759	PV230686	PV153728	PV158017
**Bibionidae**								
*Bibiomarci* (Linnaeus, 1758)	OUT1	Czech Republic, 2013	KJ136689	KJ136724	KP288758	KJ136761	KT316846	KX453730
*Dilophusfemoratus* Meigen, 1804	OUT23	Slovakia, 2014	KP288696	KP288728	KP288773	KP288811	KT316864	KX453740
*Penthetriafunebris* Meigen, 1804	OUT14	Slovakia, 2014	KP288689	KP288721	KP288767	KP288804	KT316858	KX453735
*Plecianearctica* Hardy, 1940	OUT2	USA, 2013	KJ136690	KJ136725	KP288759	KJ136762	KT316847	n/a
**Bolitophilidae**								
*Bolitophilacinerea* Meigen, 1818	B1	Slovakia, 2012	–	KJ136712	–	–	–	–
	BolCin	Czech Republic, 2016	ON601484	–	ON650659	ON601118	MT446881	ON706250
*Bolitophilahybrida* (Meigen, 1804)	BolHyb	Slovakia, 2021	JANSTU000000000.1					
**Canthyloscelidae**								
*Canthyloscelispectipennis* Edwards, 1930	CanPic	Argentina, 2022	PV156346	PV156355	PV206757	PV230685	PV153726	n/a
*Hyperoscelisveternosa* Mamaev & Krivosheina, 1969	OUT24	Slovakia, 2014	KP288697	KP288729	KP288774	KP288812	KT316865	KX453741
*Synneuronannulipes* Lundstrom, 1910	OUT57	Slovakia, 2016	KX453693	KX453701	n/a	KX453713	KX453763	n/a
**Cecidomyiidae**								
*Asphondyliasarothamni* (Loew, 1850)	OUT18	Czech Republic, 2014	KP288692	KP288724	KP288770	KP288807	KX453761	KX453738
*Catochaangulata* Jaschhof, 2009	C26	Slovakia, 2014	KP288677	KP288711	KP288750	KP288792	KT316837	KX453719
*Catotrichasubobsoleta* (Alexander, 1924)	OUT42	USA, 2014	KP288706	KP288738	KP288784	KP288821	KT316873	KX453747
*Porricondylanigripennis* (Meigen, 1880)	OUT16	Slovakia, 2014	KP288690	KP288722	KP288768	KP288805	KT316859	KX453736
**Ceratopogonidae**								
*Culicoidessonorensis* Wirth & Jones, 1957	culson	from GB	BK065013	BK065013	n/a	n/a	BK065013	GAWM00000000.1
**Chaoboridae**								
*Chaoborusflavicans* (Meigen, 1830)	CH-F2	Slovakia, 2019	PV156348	PV156356	PV206758	n/a	PV153727	PV158016
**Chironomidae**								
*Cricotopusdraysoni* Cranston & Krosch, 2015	cridra	from GB	GFNI00000000.1	GFNI00000000.1	n/a	n/a	GFNI00000000.1	GFNI00000000.1
*Chironomustepperi* Skuse, 1889	chitep	from GB	NC_016167	NC_016167	KC177280	KC177658	NC_016167	FJ040616
**Culicidae**								
*Anophelesgambiae* Giles, 1902	anogam	from GB	NC_002084	NC_002084	AM157179	KC177663	NC_002084	KC177121
**Deuterophlebiidae**								
*Deuterophlebiacoloradensis* Pennak, 1945	deucol	from GB	n/a	n/a	GQ465776	FJ040539	GQ465781	KC177114
**Diadocidiidae**								
*Diadocidiaferruginosa* (Meigen, 1830)	SJ1	Slovakia, 2010	–	MG554126	KP288786	–	–	–
	diafer	Czech Republic, 2016	PV156347	–	–	ON601121	MT446885	PV158015
*Diadocidiaglobosa* Papp & Ševčík, 2005	SJ9	Thailand, 2008	KP288708	KP288740	KP288789	KP288822	KT316878	KX453755
**Ditomyiidae**								
*Ditomyiafasciata* (Meigen, 1818)	SJ3	Czech Republic, 2010	KJ136698	MG554125	MG554141	KJ136770	MG554168	KX453753
*Symmerusannulatus* (Meigen, 1830)	D2	Slovakia, 2012	MT446483	KX453696	–	KX453708	KX453757	–
	symann	from GB	–	–	FJ171934	–	–	KC177112
**Dixidae**								
*Dixasubmaculata* Edwards, 1920	OUT58b	Czech Republic, 2015	KX453694	KX453702	KX453706	KX453714	KX453764	–
	dixsub	from GB	–	–	–	–	–	KC177123
**Hesperinidae**								
*Hesperinusbrevifrons* Walker, 1848	OUT41	USA, 2014	KP288705	KP288737	KP288783	KP288820	KT316872	KX453746
*Hesperinusninae* Papp & Krivosheina, 2010	OUT12	Georgia, 2013	KP288687	KP288719	KP288765	KP288802	KT316856	KX453734
**Keroplatidae**								
*Keroplatustestaceus* Dalman, 1818	B7	Slovakia, 2014	KJ136683	MG554129	KP288746	–	–	KX453716
	KerTes	Czech Republic, 2016	–	–	–	ON601130	MT446947	–
*Lygistorrhinasanctaecatharinae* Thompson, 1975	lygsan	USA, 2018	–	MT446624	PV206760	ON601102	MT446948	PV158018
	K105	USA, 2018	MT446547	–	–	–	–	–
*Macroceracentralis* Meigen, 1818	K10	Slovakia, 2013	KP288682	KP288716	KP288755	KP288797	KT316841	KX453723
*Paleoplatyurajohnsoni* Johannsen, 1910	PaJo	Italy, 2016	MT446551	MT446627	PV206761	MT446846	–	–
	K80	Italy, 2016	–	–	–	–	MG049755	MT446675
**Limoniidae**								
*Rhipidiasejuga* Zhang, Li & Yang, 2014	rhisej	from GB	GEMJ00000000.1					
**Mycetophilidae**								
*Exechiafusca* (Meigen, 1804)	ExFu	Czech Republic, 2016	MG684498	MH114203	MG684611	ON601123	MG684785	–
	E19	Czech Republic, 2015	–	–	–	–	–	MK133002
*Gnoristebilineata* Zetterstedt, 1852	GS4	Czech Republic, 2009	KP288679	KP288713	KP288752	KP288794	KT316839	KX453720
*Sciophilarufa* Meigen, 1830	SciRuf	Czech Republic, 2021	MT446554	ON601475	PV206765	ON601116	MT446956	PV158021
*Taxicnemismarshalli* Matile, 1989	NZ1	New Zealand, 2016	ON601489	ON601459	ON650665	ON601136	ON601531	ON706255
**Pachyneuridae**								
*Cramptonomyiaspenceri* Alexander, 1931	OUT37	USA, 2014	–	–	KP288779	–	–	–
	craspe	from GB	NC_016203	NC_016203	–	KC177653	NC_016203	FJ040632
*Pachyneurafasciata* Zetterstedt, 1838	OUT40	Finland, 2012	KP288704	KP288736	KP288782	KP288819	KT316871	KX453745
**Psychodidae**								
*Clogmiaalbipunctata* (Williston, 1893)	OUT21	Czech Republic, 2014	KP288695	KP288727	–	KP288810	KT316863	–
	cloalb	from GB	–	–	KC177281	–	–	FJ040622
**Ptychopteridae**								
*Ptychopteraalbimana* (Fabricius, 1787)	OUT51	Czech Republic, 2015	KX453691	KX453699	KX453704	KX453711	KX453762	KX453750
*Ptychopteralacustris* Meigen, 1830	PtyLa	Czech Republic, 2018	PV156351	PV156359	PV206763	PV230688	PV153730	PV158020
**Scatopsidae**								
*Aspistesberolinensis* Meigen, 1818	OUT43	Czech Republic, 2013	KP288707	KP288739	KP288785	KX453710	–	–
	AspBer	Czech Republic, 2018	–	–	–	–	PV153725	PV158014
*Scatopsenotata* (Linnaeus, 1758)	OUT3	Czech Republic, 2011	KJ136691	KJ136726	KP288760	KJ136763	KT316848	KX453731
**Sciaridae**								
*Bradysiatilicola* (Loew, 1850)	bratil	from GB	GQ387651	GQ387651	KC177279	FJ040522	GQ387651	FJ040621
*Sciarahemerobioides* (Scopoli, 1763)	SciHem	Czech Republic, 2018	MT446553	MT446628	PV206764	PV230689	MT446955	MT446687
**Simuliidae**								
*Parasimuliumcrosskeyi* Peterson, 1977	parcro	from GB	AF049472	n/a	n/a	KC177660	FJ524493	KC177118
**Tanyderidae**								
*Protoplasafitchii* Osten Sacken, 1859	profit	from GB	KC177472	KC177462	KC177286	KC177670	NC_016202	FJ040626
**Thaumaleidae**								
*Thaumaleabezzii* Edwards, 1929	OUT53	Slovakia, 2015	KX453692	KX453700	KX453705	KX453712	–	KX453751
	thabez	from GB	–	–	–	–	KT215925	–
**Tipulidae**								
*Tanypteraatrata* (Linnaeus, 1758)	TanAtr	Czech Republic, 2021	PV156353	PV156361	PV206767	PV230691	PV153732	n/a
*Tipulaabdominalis* (Say, 1823)	tipabd	from GB	KC177466	KC177457	KC177288	KC177678	KC192958	GQ265584
**Trichoceridae**								
*Trichocerasaltator* Harris, 1778	trisal	from GB	GAXZ00000000.2					
**Sciaroidea *incertae sedis***	–	–	–	–	–	–	–	–
*Heterotrichatakkae* Chandler, 2002	HetTak	Greece, 2016	MG684499	MG684543	MG684612	ON601125	MG684786	MH114336
*Chiletrichaspinulosa* Chandler, 2002	MJ16	Chile, 2000	KT316809	KT316814	KT316819	KT316824	KX453760	KX453727
*Insulatrichahippai* Jaschhof, 2004	IS4a	New Zealand, 2016	ON601487	ON601480	ON650663	ON601128	ON601501	ON706253
*Ohakuneabicolor* Edwards, 1927	MJ38	New Zealand, 2002	KT316810	KT316815	KT316820	KT316825	KT316844	KX453728
*Rangomaramatonnoiri* Jaschhof & Didham, 2002	MJ51	New Zealand, 2002	ON601488	ON601481	ON650664	ON601135	ON601502	–
*Rangomarama* sp.	MJ54	New Zealand, 2002	–	–	–	–	–	ON706254
*Sciaropotajaponica* Chandler, 2002	DL30	South Korea, 2022	OQ533498	OQ533499	OQ533501	OQ533500	OQ525969	OQ539524
**Brachycera**								
*Asiluscrabroniformis* Linnaeus, 1758	asicra	from GB	KC177475	KC177451	KC177289	KC177704	KC192962	EF650383
*Bombyliusmajor* Linnaeus, 1758	bommaj	from GB	KC177474	KC177450	KC177290	KC177708	KC192961	KC177144
*Haematopotapluvialis* Linnaeus, 1758	haeplu	from GB	KC177479	KC177454	KC177294	KC177694	MZ563333	n/a
*Muscadomestica* Linnaeus, 1758	mucdom	from GB	AY573084	KC347601	KC177313	KC538816	JX438043	AY280689
*Platypezaanthrax* Loew, 1870	plaant	from GB	GCGU00000000.1	n/a	n/a	GCGU00000000.1	GCGU00000000.1	n/a
*Xylomyamaculata* (Meigen, 1804)	XylMac	Czech Republic, 2020	PV156354	PV156362	PV206768	PV230692	PV153733	n/a

## Supplementary materials

Supplementary material 1 Linked Data table of specimens used for the phylogenetic analysis Data type xlsx Explanation note File includes linked table with taxa used in the phylogenetic analysis and GenBank accession numbers for sequences. https://binary.pensoft.net/file/1322779This dataset is made available under the Open Database License (http://opendatacommons.org/licenses/odbl/1.0/). The Open Database License (ODbL) is a license agreement intended to allow users to freely share, modify, and use this Dataset while maintaining this same freedom for others, provided that the original source and author(s) are credited.Alexei Polevoi, Nikola Burdíková, Jan Ševčík
